# Isolated Catatonia as a Rare Initial Manifestation of Moyamoya Disease

**DOI:** 10.7759/cureus.81791

**Published:** 2025-04-06

**Authors:** Ouafae El Hayani, Bnouhanna Wadii, Mounia Rahmani, Maria Benabdeljlil, Firdaous Touarsa, Meriem Fikri, Saadia Aïdi

**Affiliations:** 1 Neurology, Specialty Hospital, Ibn Sina University Hospital Center, Mohammed V University, Rabat, MAR; 2 Neurology, Avicenne University Hospital, Rabat, MAR; 3 Neurology and Neuropsychology, Specialties Hospital of Rabat, Avicenne University Hospital, Rabat, MAR; 4 Neurology and Neuropsychology, Faculty of Medicine and Pharmacy, Specialty Hospital, Mohammed V University, Rabat, MAR; 5 Radiology, Ibn Sina Teaching Hospital, Rabat, MAR; 6 Neuroradiology, Specialty Hospital, Rabat, MAR

**Keywords:** catatonia, cerebral mri, moyamoya disease, moyamoya syndrome, psychiatric disorders

## Abstract

Moyamoya disease (MMD) is a rare, idiopathic, and life-threatening cerebrovascular disorder characterized by progressive stenosis and occlusion of the intracranial internal carotid arteries, leading to the formation of abnormal collateral vessels. The incidence of MMD is high in Asian countries but has also been reported in other regions with lower prevalence. Clinically, the disease can manifest with ischemic, hemorrhagic, or epileptic symptoms. Psychiatric manifestations are atypical and may be mistaken for primary psychiatric disorders, necessitating a specialized approach to management. We report a rare clinical case of a patient who was admitted with isolated catatonia and was diagnosed with MMD through neuroimaging. We discuss the possible mechanisms underlying this association, radiological findings, and strategies for managing such an uncommon presentation.

## Introduction

Moyamoya disease (MMD) and moyamoya syndrome (MMS) are rare, progressive cerebrovascular disorders affecting the circle of Willis. They involve the distal segments of the internal carotid arteries and/or the proximal portions of the anterior and middle cerebral arteries, leading to the development of collateral vessels [1]. The prevalence of MMD is highest in East Asia [1], with reported rates of 10.9 and 16.1 per 100,000, respectively [1].

The incidence of MMD follows a bimodal distribution, with peaks in the first and fourth decades of life [2], and it is associated with significant morbidity and mortality [3]. When the disease occurs without an identifiable cause, it is referred to as MMD. However, when associated with conditions such as neurofibromatosis type 1, Down syndrome, prior head irradiation, or sickle cell disease, it is classified as MMS [3].

In pediatric patients, MMD most commonly presents with transient ischemic attacks or ischemic strokes, whereas in adults, hemorrhagic strokes are more frequent [4]. Isolated neuropsychiatric manifestations have been rarely reported in association with MMD [5]. These manifestations likely result from chronic cerebral hypoperfusion, which can affect specific brain regions even in the absence of stroke [6].

We describe a rare and challenging case of a woman who presented with catatonia and was ultimately diagnosed with MMD.

## Case presentation

We report a case of a 45-year-old right-handed woman of Moroccan origin, with a history of primary infertility but without psychiatric history, who presented to the neurological emergency department with sudden-onset mutism accompanied by behavioral disturbances, including postural maintenance and stupor, which had lasted for one week prior to admission. Two days following the onset of these symptoms, the patient experienced two generalized tonic-clonic seizures.

On admission, the patient was afebrile and normotensive, with a normal pulse and respiratory rate. Physical examination revealed a patient with a fixed gaze, postural maintenance, negativism, catalepsy, mutism, and stupor, meeting the Diagnostic and Statistical Manual of Mental Disorders, Fifth Edition (DSM-5) criteria for catatonic syndrome [7]. The rest of the neurological examination was unremarkable, with present and symmetrical osteotendinous reflexes. She exhibited spontaneous movement of all four limbs. Sensitivity, coordination, and language could not be evaluated due to the stuporous state of the patient.

**Figure 1:**
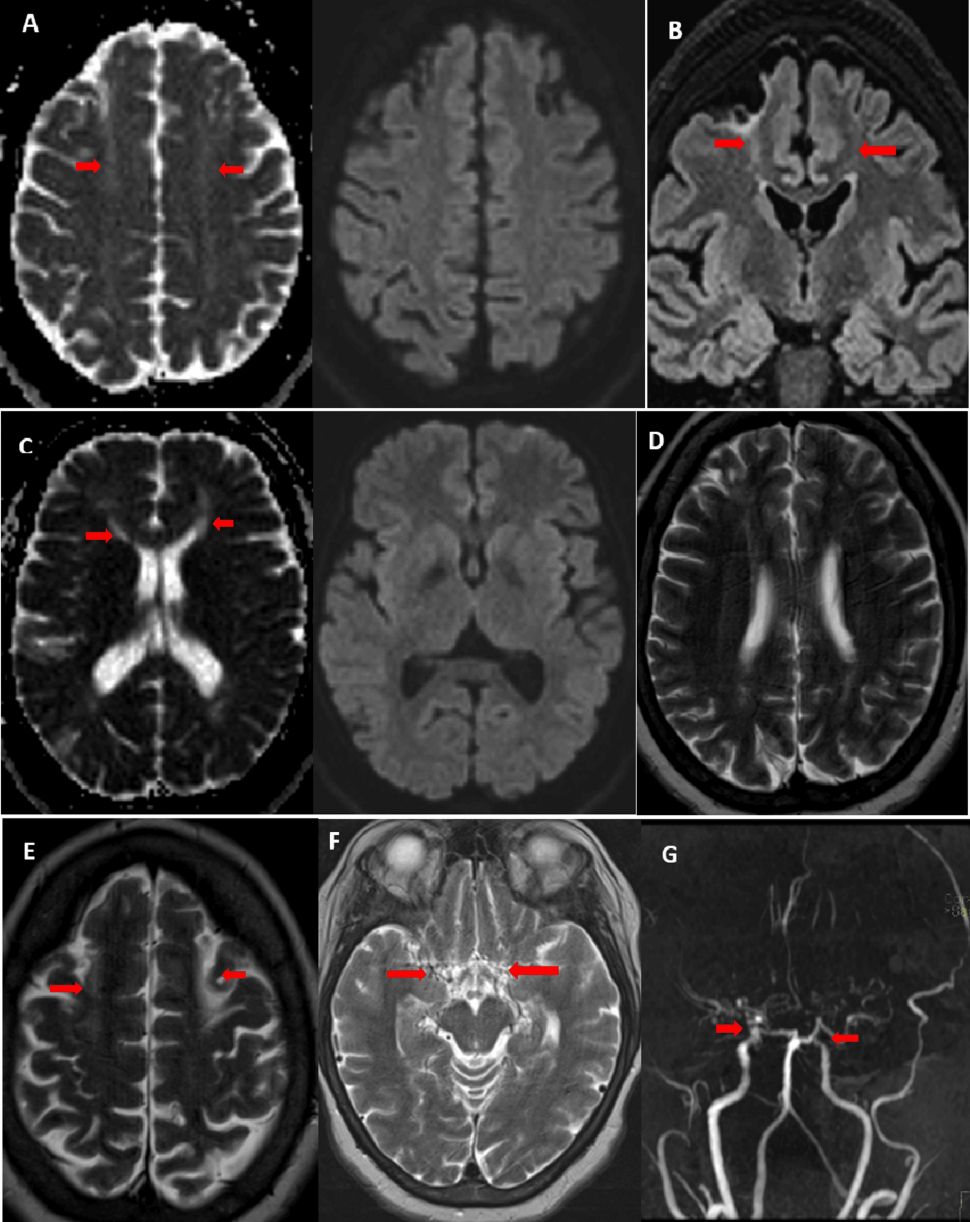
Cerebral MRI showing bilateral frontal cortico-subcortical hyperintensities on apparent diffusion coefficient (ADC) with normal diffusion (A, C), fluid-attenuated inversion recovery (FLAIR) (B), and T2 (D, E) sequences. Magnetic resonance angiography (MRA) of the circle of Willis (G) demonstrates internal carotid artery stenosis bilaterally, with absence of visualization of the middle cerebral arteries (MCA) and anterior cerebral arteries (ACA), replaced by a moyamoya collateral network visible on the T2 sequence (F).

**Figure 2:**
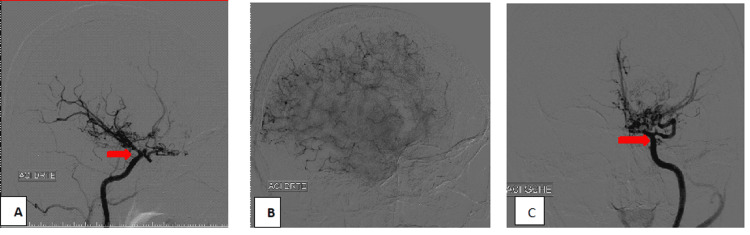
Cerebral arteriography showing bilateral occlusion of the internal carotid arteries: (A) right internal carotid artery, (C) left internal carotid artery, with a moyamoya network forming the characteristic "puff of smoke" appearance (B).

An electroencephalogram was normal, while brain MRI showed bilateral junctional cortico-subcortical frontal hyperintensities on T2 and fluid-attenuated inversion recovery (FLAIR) sequences. Magnetic resonance angiography (MRA) revealed bilateral stenosis of the internal carotid arteries, indicative of moyamoya syndrome (Figure 1). Cerebral arteriography further confirmed the diagnosis by demonstrating a harmonious reduction in the caliber of both the right and left cervical and intracranial internal carotid arteries, with an absence of opacification of segments A1 and M1, and the development of a fine anastomotic arteriolar network resembling moyamoya at the base of the skull bilaterally (Figure 2).

A comprehensive etiological assessment was conducted, including a normal dermatological examination and normal laboratory tests (thyroid panel, blood smear, and hemoglobin electrophoresis) (Table 1).

**Table 1:** Patient's laboratory results and reference values.

Assessment type		Result	Unit	Reference range
Hemoglobin (HB)		12.8	g/dl	12.0-15.5 g/dl
Mean corpuscular volume (MCV)		82.2	μm^3^	79-99 μm^3^
Mean corpuscular hemoglobin concentration (MCHC)		33	%	32-36%
White blood cells (WBC)		6	10⁹/L	4-10 ×10⁹/L
Platelets (PLT)		290	10⁹/L	150-400 × 10⁹/L
Aspartate aminotransferase		17	UI/L	5-34 UI/L
Alanine aminotransferase		22	UI/L	0-55 UI/L
Ultrasensitive thyroid-stimulating hormone		1.2	UI/L	0.4-4 mUI/L
Hemoglobin electrophoresis	Adult hemoglobin (HB A)	97.8	%	96-98%
	HB A2	2	%	2-3%
	Fetal hemoglobin (HB F)	0.2	%	<1%
Urea		0.27	g/l	0.15-0.55 g/l
Creatinine		7	Mg/l	5.7-12.5 mg/l

The patient was treated with lorazepam 2.5 mg, half a tablet three times daily, resulting in a 50% regression of catatonic symptoms after the first dose. Consequently, the dosage was increased to one tablet three times daily, leading to the complete resolution of catatonia. However, this was followed by behavioral disturbances such as psychomotor agitation and aggression, necessitating the initiation of quetiapine, which resulted in a favorable clinical outcome. Surgical treatment was not proposed due to a lack of expertise in Morocco. At follow-up, the patient remained clinically stable, with no recurrence of catatonic symptoms or seizures. Neurological and psychiatric evaluations performed at three and six months post discharge were unremarkable.

## Discussion

MMD is characterized by progressive steno-occlusion of the intracranial arteries, particularly the terminal portion of the internal carotid artery, in the absence of other identifiable causes [8,9]. Unlike previous definitions, the condition does not necessarily require bilateral involvement of the intracranial carotid artery [3]. In some cases, unilateral MMD may evolve into bilateral distal internal carotid artery occlusive lesions, a pattern more commonly seen in children than in adults [10].

MMD has the highest prevalence in East Asia, particularly in Japan, Korea, and China, with a female predominance. Cases have also been reported in North America and Europe, though at a lower prevalence [1]. Data from the United States remains limited, although an evaluation of 2,280 MMD-related hospital admissions indicated an annual prevalence rate of 0.57 per 100,000 individuals [1,11]. Another US-based estimate reported an incidence rate of 0.086 per 100,000 person-years [11,12]. In European countries, MMD is significantly less common, with an incidence of approximately three cases per million, which is roughly one-tenth the rate observed in Japan [4].

Approximately 15% of cases have a familial component. The RNF213 gene, located on chromosome 17q25.3, is the most strongly associated [11].

MMD is rare, and in Morocco, there is no registry available to determine its prevalence. A search on PubMed and Google Scholar returned fewer publications, reporting a total of around 12 cases.

The term "Moyamoya," originally described by Suzuki and Takaku in 1969 as "bilateral hypoplasia of the internal carotid arteries" [2], derived from Japanese meaning "puff of smoke," describes the angiographic appearance of collateral vessels formed in response to arterial stenosis in the distal internal carotid arteries, proximal anterior, and middle cerebral arteries [1]. Involvement of the posterior cerebral artery is rare but is associated with a worse prognosis [1].

Radiological evaluation is essential for diagnosing MMD or MMS. It reveals varying degrees of stenosis or occlusion affecting the large arteries in the anterior part of the circle of Willis, along with the development of collateral vessels, which create a characteristic angiographic appearance known as the "clouds of smoke" or "puff of cigarette smoke" [2], as observed in our patient. Collateral circulation arises from the dilation of existing arteries or the formation of new perforating vessels [2].

Digital subtraction angiography (DSA) is the gold standard for diagnosing MMD or MMS [1]. Current diagnostic criteria from the Research Committee on Moyamoya Disease (RCMD) also support the use of MRI and MRA in specific cases (Table 2) [8,9]. These imaging modalities can detect complications such as hemorrhage and ischemia, as well as leptomeningeal collateral recruitment ("Ivy sign"), and they are valuable for sequential follow-up examinations [3].

**Table 2:** Diagnostic criteria for moyamoya disease (2021). References [8,9]. MRA: magnetic resonance angiography.

Criteria
A. Radiological observations
1. Cerebral angiography
(a) Narrowing or blockage detected in the distal segment of the intracranial internal carotid artery.
(b) Presence of abnormal vascular formations, known as moyamoya vessels, near the areas of stenosis or occlusion—these appear during the arterial phase of the angiographic study.
Important: Diagnosis can be made in both bilateral and unilateral presentations.
2. Cerebral MRI and angio-MRI imaging
(a) Narrowing or blockage at the distal end of the intracranial internal carotid artery.
(b) A bilateral reduction in the external diameter of the terminal internal carotid arteries and the middle cerebral arteries observed on T2-weighted MRI scans.
(c) Detection of atypical vascular networks within the basal ganglia and/or the periventricular white matter on MRA.
Important: MRI/MRA should be performed using equipment with a static magnetic field strength of at least 1.5 Tesla.
B. Other diagnoses: The following conditions should be ruled out
(1) Autoimmune disorders (systemic lupus erythematosus, antiphospholipid syndrome)
(2) Meningitis
(3) Intracranial tumors
(4) Down syndrome
(5) Neurofibromatosis type 1
(6) Cerebrovascular damage due to prior cranial radiation therapy
(7) Moyamoya disease may still be diagnosed in individuals with hyperthyroidism
Important: A diagnosis of moyamoya disease is confirmed when either criteria (a) and (b) under section A-1, or all three criteria under section A-2 are satisfied, and the conditions listed in section B have been excluded.
The classifications “definite case” and “probable case” were removed from the diagnostic guidelines in the 2015 revision.

The Suzuki classification is used to assess the stage of MMD based on angiographic changes in collateral vessel formation over time [13]. It defines six stages of severity: in stage 1, there is only carotid artery stenosis; in stages 2 and 3, moyamoya-type collateral vasculature develops and expands; in stages 4 and 5, these vessels start to regress; and by stage 6, they become completely absent [2]. Our patient meets the criteria for stages 2 and 3. The Berlin grading system has recently been found to be superior to the traditional Suzuki staging system [1].

Diagnosing MMD becomes more challenging in the later stages described by Suzuki, as the disappearance of moyamoya vessels complicates detection [1]. Additionally, MMD can be mistaken for intracranial atherosclerotic disease or vasculitis [14], particularly in the early stages when moyamoya vessels are not yet visible [3].

MMD presents differently across age groups. In children, the most common symptoms are transient ischemic attacks or ischemic strokes, while adults more frequently experience hemorrhagic strokes [4]. Other neurological symptoms include sudden-onset focal deficits, headaches, vertigo, seizures, involuntary movements [2], altered consciousness, and neurocognitive impairment [15]. MMD manifests in four clinical forms: ischemic, hemorrhagic, epileptic, and other [16].

In European populations, the onset appears to occur later than in Asian populations and is associated with a lower incidence of hemorrhagic events [2].

Isolated psychiatric presentations of MMD are rare. While no exact prevalence has been established in the literature, available studies and case reports suggest that such manifestations are highly uncommon. In most documented cases, psychiatric symptoms tend to emerge alongside neurological complications such as stroke, intracranial hemorrhage, or cognitive decline [1,4,12,17]. Psychiatric comorbidities in MMD are not uncommon overall. In one large study, nearly 39% of patients were found to suffer from psychiatric disorders, most commonly depression (13.5%) and anxiety (8.0%) [17]. Another study observed anxiety in 32.7% of patients and depression in 29.5%, with women more frequently affected [1]. In patients who experienced hemorrhagic forms of MMD, depression was present in up to 73% of cases [4]. Emotional suffering, including anxiety and depressive symptoms, was reported in over a third (37%) of patients, and nearly a quarter (23%) showed measurable cognitive impairments on neuropsychological testing [4].

In children, the clinical picture may differ. Rather than diagnosed mood disorders, they may present with emotional instability, hyperactivity, and attention difficulties [11].

Among all these neuropsychiatric symptoms, catatonia stands out as particularly rare, with only a few cases described in the literature [15].

Only two cases of isolated catatonia as a manifestation of MMD have been reported [13]. The first case, described by Ghignone et al. in 2015, involved a 30-year-old woman with sickle cell disease who developed catatonia. Cerebral MRI and MRA revealed stenosis of both the left and right internal carotid arteries (ICAs), along with a decreased caliber of the right posterior cerebral artery and multiple collaterals adjacent to both middle cerebral arteries, consistent with MMS. She was successfully treated with electroconvulsive therapy (ECT) [18].

The second case, reported by Lai et al. in 2018, involved a 34-year-old Chinese woman who developed catatonia following a hemorrhagic stroke event. This was attributed to cognitive impairments resulting from a history of intraventricular hemorrhages [4]. Her MRI showed a right temporal hemorrhagic stroke and a left sequelae hemorrhagic stroke. Angiography revealed stenosis of the distal intracranial carotid artery at the supraclinoid segment, along with a dense network of abnormally dilated collateral vessels, creating the characteristic "puff of smoke" appearance.

A 2024 meta-analysis by Saccaro et al. identified 29 cases of MMS with psychiatric manifestations, with an average diagnostic age of 23 years and a predominance of female patients [15]. In 19 cases, psychiatric symptoms preceded the diagnosis of MMD, with anxiety and depression being the most common. However, catatonia is rare, with only two cases already described above. Our case is therefore the third to be described.

The association between catatonic syndrome and MMS or MMD is poorly understood, and likely has a multifactorial pathogenesis [17], possibly due to the presence of microstructural vascular lesions in the frontal white matter secondary to chronic hypoperfusion [6]. Chronic cerebral hypoxia secondary to hypoperfusion may be sufficient to cause clinical repercussions without the need for infarction [6]. Calvière et al. measured the ADC and cerebrovascular reserve (CVR) in MMD patients with normal-appearing frontal white matter on MRI and concluded that elevated ADC, reduced CVR, and executive dysfunction were observed [19].

It has been reported that the reduction in CVR is more pronounced in children, as assessed through tests involving acetazolamide or CO2. These differences may explain the higher incidence of ischemic lesions in pediatric populations, as opposed to the more frequent occurrence of intracranial hemorrhages in adults [2,20].

Psychotic symptoms were more frequently observed in patients with predominant left hemisphere involvement, suggesting a relationship between MMD localization and psychiatric manifestations. The left hemisphere is heavily involved in language, reasoning, attention, and semantic memory. Therefore, a lesion or hypoperfusion in this area may contribute to cognitive deterioration and derealization, often observed in individuals with psychosis [15]. A lesion in Broca’s area or the left temporo-parietal junction leads to judgment impairments and delusional ideas, characteristic of psychotic thinking. Functional MRI studies have revealed the involvement of several brain structures in the genesis of psychosis, particularly the thalamus [15]. One study found that frontal lobe involvement in MMD or MMS is correlated with paranoia [1], while another study found that depression in MMD or MMS is significantly associated with damage to the right non-dominant hemisphere in the middle cerebral artery region [1].

Conventional MRI alone may not have been sufficient. MRA provides a non-invasive, highly sensitive method to visualize intracranial vasculature, allowing for the detection of stenosis or occlusion in the distal internal carotid arteries and the presence of abnormal collateral networks characteristic of moyamoya [5,11]. However, widespread screening of all psychiatric patients with MRA is not currently justified, given the low prevalence of MMD and the cost and accessibility constraints of advanced neuroimaging. Instead, a more pragmatic approach would involve targeted MRA. Richards et al. emphasize that an MRA should be requested in the presence of atypical psychiatric features, such as new-onset or rapidly progressing psychiatric symptoms, absence of a family history of psychiatric disorders, family history or ethnic backgrounds associated with higher MMD risk or the presence of visual hallucinations, co-existing subtle neurological signs or cognitive decline [11,15], and seizures or episodes of altered consciousness without a clear psychiatric explanation. This was precisely the case with our patient, where the MRA was crucial in establishing the diagnosis. Similarly, Llunell-Paz et al. highlight that treatment resistance in psychiatric disorders is a red flag that should prompt consideration of alternative diagnoses, such as MMD [6].

Treatment with benzodiazepines is commonly described for managing MMD-related anxiety and agitation, but their efficacy as monotherapy has been reported to be poor due to rapid tolerance and possible paradoxical disinhibition. They are preferred for short-term management of anxiety or emergent agitation [11]. The effectiveness of benzodiazepines, particularly lorazepam, in treating catatonic syndrome, especially of neurological origin, has been well established [12]. The "benzodiazepine test" involves administering a dose of 1 to 2 mg of lorazepam, either orally or intravenously, and then reassessing the patient after about 30 minutes. A 50% reduction in clinical symptoms, such as immobility, mutism, or rigid posturing, is generally considered a good indicator of response to treatment, as demonstrated in our patient. This approach helps confirm that the syndrome is indeed catatonic and not related to another psychiatric or medical condition [12].

ECT represents a first-line treatment in emergency situations and can save lives in cases of extreme negativism or malignant catatonia [4]. However, the rapid increase in blood pressure and cerebral blood flow associated with ECT could potentially increase the risk of hemorrhagic stroke. Nonetheless, two cases in the literature have described ECT being administered safely with proper precautions.

In the majority of case reports, it has been noted that psychotic symptoms improved after antipsychotic treatment (particularly with risperidone and clozapine), suggesting that these medications provide effective symptomatic treatment [15]. Although no controlled trials have evaluated the effectiveness of these medications in managing associated psychiatric disorders, initiating psychotropic treatment in patients with MMD must consider its impact on cerebral perfusion, epileptogenic threshold, the potential for paradoxical disinhibition, and disease characteristics (such as the exact location of occlusions and the presence of hemorrhages). Clozapine should not be used as a first-line treatment due to its side effects, and paliperidone, being an active metabolite of risperidone, is also expected to be effective [15].

Patients with MMD exhibit a heightened sensitivity to neuroleptics, frequently presenting with extrapyramidal symptoms and neuroleptic malignant syndrome, particularly when treated with first-generation antipsychotics [21]. Second-generation agents, such as quetiapine, are preferred due to their lower risk of extrapyramidal complications [11].

## Conclusions

MMD is rare, and its isolated psychiatric manifestations are even more uncommon. Among these, catatonic syndrome as a presenting feature is exceptionally rare, with our case being only the third reported in the literature. This unique scenario highlights the necessity of conducting both parenchymal and vascular MRI in cases of atypical psychiatric presentations, as MMS has been documented to manifest as an isolated catatonic syndrome. This case shows the importance of ongoing multidisciplinary follow-up. Given the progressive nature of MMD and the risk of both ischemic and hemorrhagic events, long-term monitoring remains essential to promptly identify and manage potential recurrences or complications.
